# Mindlessly Polite: A Conceptual Replication of the Emotional Seesaw Effect on Compliance and Information Processing

**DOI:** 10.3389/fpsyg.2017.00239

**Published:** 2017-02-21

**Authors:** Magdalena C. Kaczmarek, Melanie C. Steffens

**Affiliations:** ^1^Department of Psychosocial Medicine and Psychotherapy, University Hospital, Friedrich-Schiller-University JenaJena, Germany; ^2^International Max Planck Research School of Adaptive Behavior in a Fundamentally Uncertain World, Max Planck Institute of EconomicsJena, Germany; ^3^Department of Social and Economic Psychology, University Koblenz-LandauLandau, Germany

**Keywords:** emotional seesaw phenomenon, contrasting emotions, social influence technique, compliance, cognitive processing

## Abstract

Recent studies demonstrated that the sequential induction of contrasting negative and positive emotions can be used as a social influence technique. The original field experiments found that whenever a sudden change in the emotional dynamic occurs – from negative to positive or vice versa – an increase in compliant behavior and an impairment in cognitive functioning can be observed. The goal of the present experiments was a conceptual replication and extension of the results in a more controlled and counterbalanced fashion. To this aim a novel emotion induction technique was created using an outcome related expectancy violation to induce and change emotions. In a first experiment, the influence of contrasting emotions (vs. only one emotion) on compliance, message processing and information recall was assessed among 80 undergraduate students. We were able to show that a positive, then negative experience, and vice versa, led to losses in processing efficacy, not only leaving individuals momentarily vulnerable to social influence attempts, but also impairing information recall. We replicated this pattern of findings in a second experiment (*N* = 41). The implications of this innovative induction technique and its findings for theory and future research on the emerging field on contrasting emotions as social-influence techniques are discussed.

## Introduction

How does experiencing a negative event that then turns out to be positive, or experiencing a positive event that then turns out as negative, influence cognitive processing and compliant behavior? The influence of emotions on human behavior (Baumeister et al., 2007), decision making (Lerner et al., 2015), and cognitive processing (Mueller, 2011) has been extensively studied. This influence though was predominantly examined using a one-directional and non-repetitive approach, testing how an individual feels or reacts to only one kind of emotional stimulus, either a positive or a negative one (Hareli and Rafaeli, 2008). However, everyone who just managed to avoid a car accident (fear-then-relief), or picked up a potential treasure from the street that turned out to be trash (happiness-then-disappointment) can relate to the idea that sequential contrasting emotions can affect us on a day-to-day basis. Yet these situations and their consequences are poorly captured by existing research on emotion.

There is reason to believe that the sequential experience of positive and negative emotions makes people more susceptible to persuasive attempts than the experience of either type of emotion alone – an idea originally proposed by Dolinski and Nawrat (1998). These authors showed that contrasting emotions impair some aspects of cognitive processing and can be used as tools of social influence. The researchers called this new social influence technique the emotional seesaw phenomenon (ESP)^1^, thus extending what they previously referred to as the fear-then-relief technique (e.g., Dolinski and Nawrat, 1998). The present experiments provide an overarching conceptual replication and develop a new properly counterbalanced method of emotion induction suitable for laboratory testing. Our goal is to validate previous findings, to extend them by examining the ESP’s influence on information recall, and to provide an easy to implement method to stimulate and guide future research on contrasting emotions as social influence technique.

In everyday life, humans are faced with situations where they are talked into subscribing, acquiring, signing, and donating – behaviors that often are not compatible with their current beliefs nor needs. These situations are designed to persuade people to act without the hard work of attitude change. Although these techniques differ in many aspects from each other, many of them follow a certain procedural script (Cialdini and Goldstein, 2004). Most of the well-known techniques are based on a scripted procedure composed of sequential requests: The requesting itself proceeds in stages, each of which establishes the foundation for further changes in behavior. For example, the foot-in-the-door technique (Freedman and Fraser, 1966) starts with a small request only to gain compliance with a larger target request afterward. Meta-analyses for such techniques suggest that the probability of compliance raises significantly compared to a situation where the target request is asked straight away (Burger, 1999; Pascual and Guéguen, 2005).

A fairly new social influence technique that does not share the characteristics of those mentioned above is the ESP. It aims at achieving compliance without following a sequential requesting structure. Inspired by the bad-cop–good-cop procedure - a well-known police interrogation technique – Dolinski and Nawrat (1998) investigated the influence of sequential opposing emotions on compliance and cognitive performance. The experiments entailed a mix of emotions in order to wield influence over a target person. In some studies, the researchers implemented a shift from negative to positive: They scared jaywalkers who crossed a street illegally via a police whistle, or frightened parking violators with parking tickets that turned out to be advertisements (Dolinski and Nawrat, 1998). In other studies, the emotional shift was from positive to negative, for instance, finding a banknote that turned out to be an advertisement, or informing people that due to an overpayment they would be reimbursed with a considerable sum of money and later claiming that this was a computer mistake (Nawrat and Dolinski, 2007). Several interesting findings emerged from these experiments. Among the most consistent results was that people undergoing a sequence of contrasting emotions (irrespective of the direction) showed higher susceptibility to compliant behavior. The sequence promoted not only non-consequential compliance to a senseless plea, such as switching the telephone receiver from one ear to the other in order to check the permeability of the line; it was shown as well for consequential compliance such as money donation, time spent watching a stranger’s luggage, or voluntary charity work (Dolinski and Nawrat, 1998; Nawrat and Dolinski, 2007). Some evidence also suggests that contrasting emotions lead to impairments in cognitive processing efficiency. Participants subjected to an emotional seesaw mindlessly accepted dubious requests for money donation without raising any concerns in response (Dolinski and Nawrat, 1998). This impaired cognitive appraisal of the request content was attributed to a mindless state of mind. Mindlessness, a shallow processing of information, was being hypothesized to be the reason for elevated compliance. Two additional experiments confirmed the inhibitory tendencies of contrasting emotions on cognitive processing. In the experiments, participants exhibited impairments in both simple (emotional perception) and more complex cognitive tasks (arithmetical operations) (Dolinski et al., 2002).

In essence, it can be extrapolated from the research that the sudden shift of emotions, be it from positive to negative or vice versa, induces mindlessness and in turn promotes behavioral compliance. Yet critical questions regarding causality remained empirically untouched. Our first goal was to investigate the independence of the findings from the procedures used and from the context by eliminating methodological ambiguities prior studies were unable to control. Our experiment was designed to answer the question whether the display of contrasting emotions is more powerful for impairing cognitive processing and generating compliance than the display of either only negative or only positive emotions. We assert that a properly counterbalanced conceptual replication in a controlled environment could not only validate the existing findings, but also possibly provide insight into the mechanism and further consequences. For this reason, apart from testing existing hypotheses, we also examine the influence of contrasting emotions on a different aspect of cognitive processing, namely information recall. Because prior research suggests a close connection between contrasting emotions and an inhibition in processing efficiency, we propose that memory processing should be inhibited as well.

### The Current Experiments

The current study involves an innovative way of inducing contrasting emotions in a laboratory setting. The shift from positive to negative and vice versa is induced in a parallel fashion. Because previous studies have already established that consequential compliance is increased after the ESP, we focus on a compliance scenario that does not depend on material resources, namely signing a petition. Because signing a petition should – given mindful processing – depend on ideology and content, we use a senseless request. Thus, in the presence of message elaboration, rejection should be the default option. In this regard, our approach is superior to many other compliance studies by eliminating possible compliance influencing factors such as personal resources (e.g., time or money) or ideology (e.g., attitude toward the request). This setup allows us to measure not only compliance rates but also – based on participants’ concerns or questions regarding the senseless petition – the presence or absence of message elaboration (see Dolinski and Nawrat, 1998). However, analyzing only overt behavioral measures (questions and signature), does not necessarily provide insight into processing efficiency. It could be the case that a person perfectly understood the request but was too shy to veto (no processing inhibition). This is why the main difference between the so far known consequences of contrasting emotions and our work is that, apart from measuring exclusively overt behavioral responses, we introduced analyses of memory, assessing participants’ recall of petition associated information.

We tested whether people after an emotional seesaw, compared to a control condition, showed impaired cognitive functioning, measured as message processing (H1), a greater willingness to comply (H2), and a decline in information recall (H3). Those findings should be independent of the direction of the emotional shift. Two experiments were designed to test these theoretical assumptions. The goal of the first experiment was to conceptually replicate and to extend previous findings using a new ESP induction technique based on an expectancy violating structure. The goal of the second experiment was to replicate the findings of Experiment 1 and to validate the used emotion induction procedure.

## Ethical and Methodological Considerations

Both studies were approved by the institutional review board of the Max Planck Institute of Economics. In accordance with the Declaration of Helsinki, all participants provided written consent before completing the experiment. Termination of data collection was decided in advance, based on a fixed *N*. Practical considerations determined the sample size due to the logistic effort required (at least three experimenters, two experimental rooms, and 25 min per participant). We strived for the recommended minimum of 20 observations per cell as proposed by Simmons et al. (2011). The paper is written under a full disclosure policy, all experimental treatments, collected variables along with undertaken analyses are mentioned in the text or corresponding footnotes.

## Experiment 1

### Participants

Eighty visitors of the university library in a large town in Germany voluntarily participated in the experiment. They were reimbursed with €1 and had the possibility to win €2.50, as described below. One participant was excluded from analyses because he had been overlooked by the petitionist (*N* = 79; 48 female, 31 male; *M*_age_ = 24.29, *SD*_age_ = 3.97). A *post hoc* power analyses for Chi-Square-Tests showed that this sample size allows detecting medium sized effects (*w* = 0.30), given α = 0.05, with statistical power of 1 – β = 0.76 (Faul et al., 2007). All participants were tested individually according to a prearranged random order.

### Materials and Procedure

#### Stage 1

Participants were welcomed in Experimental Room 1 (ER1). After providing informed consent, they were asked to take part in a common knowledge quiz in ER2. There they were provided with the following information: “*You will take part in a computer-based common knowledge quiz, consisting of 5 multiple-choice questions. You can win up to €2.50. Additionally you will receive a fixed participation fee of €1. After you finish the quiz please return immediately to ER1 in order to answer a questionnaire and to receive your payment. You can start the quiz now.”* Experimenters were instructed to not reveal any additional information and to leave the experimental room after the onset of the quiz to avoid questions concerning the manipulation.

#### Stage 2

In ER2, participants were randomly assigned to one of two groups: the experimental condition where an emotional seesaw was implemented (*n* = 40) vs. the control condition without emotional seesaw (*n* = 39). As a control factor, we included whether final emotional state was positive or negative. Consequently, half of the participants in each condition had a negative final emotional state and half of the participants a positive one. Emotions were manipulated by the difficulty of the questions (positive mood induction through easy questions, negative mood induction through difficult questions). Emotions were intensified by immediate feedback after each response. A payout scheme was displayed on the computer screen at the end of the game. In the control group, the proposed payout was congruent with common expectations: For every correct answer participants received €0.50, for every incorrect €0. The emotional seesaw in the experimental group was induced by an expectancy-incongruent payout scheme. The payout table was reversed: Participants received €0 for every correct answer and €0.50 for every incorrect one. As common knowledge suggests a positive monetary outcome for correct answers, the violation of those expectancies was supposed to elicit an emotional seesaw (i.e., in the case of many correct answers, the high expectation of winning much was disappointed; in the case of few correct answers, the low expectation of winning little surprisingly turned out as incorrect)^2^. The frequency of this belief and the difficulty of the questions were pretested and confirmed in a pilot study (*N* = 15).

#### Stage 3

On their way back to ER1, one of two female petitionists blind to the experimental condition approached participants with a request to sign a petition. After introducing herself as a student of the university, the petitionist asked participants to sign a petition demanding that every student should have the right to choose which public transportation to use to go to university. This is a nonsense petition because people can choose which means of transportation they want to take. Participants’ reactions constituted DV1, *Message Processing*, and DV2, *Compliance*. Petitionists were trained beforehand to display always the same behavior and use certain coding rules with three instructed dummy participants displaying different behaviors. Both petitionists were of the same gender, age, had comparable physical features (attractiveness, hair color, and length), and wore similar outfits.

##### Message processing

To test the assumption that mindless participants are less likely to ask questions or raise concerns about the nonsense request, the petitionist noted explicit questions and comments. Questions or concerns raised in response to the nonsense request were treated as indicators of conscious message elaboration. The variable was therefore coded dichotomously (message processing present vs. absent). Petitionists were instructed to classify message elaboration as present when participants indicated a basic understanding of the message (e.g., questions: “*Aren’t we already allowed to do so?*”; “*Could you elaborate on that?”*; or concerns: “*We are already allowed to choose!*”; *“This is stupid!”).* In order to circumvent possible misunderstandings due to acoustic interferences petitionists were instructed to repeat the request in the exact same manner when asked “*what*” or “*sorry.*”

##### Compliance

To test the hypothesis that people after an emotional seesaw reveal compliant behavior, participants were asked to sign a nonsense petition. The signature was treated as compliant behavior^3^.

#### Stage 4

Back in ER1 participants were asked to complete two questionnaires, one concerning the petition and the petitionist (DV3: Information Recall) and a second one concerning demographic (age, gender, and education) and control questions. To mitigate demand effects control questions included: “*What was the purpose of the petition?*”; “*Did you notice that the petition was part of the study?*”; “*What did you think this study was about?*”^4^

##### Information Recall

In order to test whether people in the seesaw group would remember less information about the confederate and the petition than people in the control group, a 9-item multiple-choice questionnaire was used, including the option “I don’t know” with each question. Example questions and response options are: *(1) Was the person who approached you: (a) male, (b) female; (2) The color of his/her hair was (a) black, (b) brunette, (c) blond, (d) red.* In order to account for guessing, an information recall index was computed based on the two-high-threshold model of recognition memory (Snodgrass and Corwin, 1988). The discrimination index (Pr) was computed by subtracting the number of wrong responses from the number of correct ones. After questionnaire completion, participants received their reimbursement, were thanked and debriefed.

### Design

The design was a between-subjects one-factor design with two levels (experimental condition: one emotion, *n* = 39, vs. emotional seesaw, *n* = 40). [Final] emotional state was included as a control factor. Dependent measures were (a) message processing, (b) compliance, and (c) information recall. To each affirmative behavior [(a) asking questions, (b) signing petition, (c) correct answer], we assigned “1” and “0” for not displaying this behavior.

### Results

**FIGURE 1:**
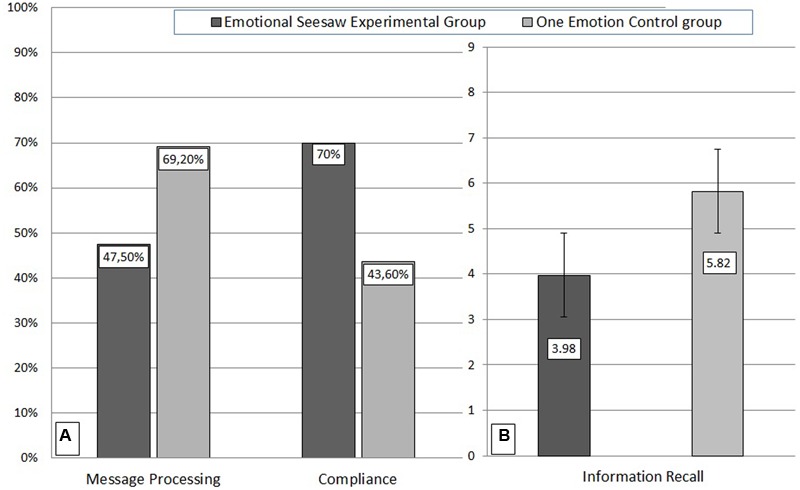
**(A)** Percentage of people who asked questions, or raised concerns in response to the petition (Message Processing), and signed the petition (Compliance). **(B)** Number of correctly recalled details concerning the petition and the petitionist minus false alarms (Information Recall).

Throughout the present paper, significance tests were conducted with α ≤ 0.05. Although all our hypotheses imply directional predictions, we report the focused one-tailed test only if the two-tailed probability is non-significant (cf. Meiser, 2011). Following established practice in social influence research (e.g., Dolinski and Nawrat, 1998; Goldstein et al., 2011) we applied Chi-Square tests to analyze proportions of explicitly stated comments in response to the nonsense petition, as well as the amount of collected signatures (see **Figure 1A**). To prevent the overestimation of statistical significance for small sample sizes we report the Yates corrected Chi-Square results. Separate results for each condition are presented in **Table 1**. Preliminary analyses including the factors age, gender, and education, as well as identity of the petitionist, yielded no statistically significant main effects or interactions. These data are therefore not presented.

**Table 1:** Frequency of Vocalized Doubts, Signatures on the Petition, and Information Remembered by Participants, Separately for each Condition.

	Message Processing		Compliance		Information Recall	
	*%*	*n/N*	*%*	*n/N*	*M*	*SD*
Positive	65	(13/20)	35	(7/20)	6.35	1.66
Negative	74	(14/19)	53	(10/19)	5.26	1.66
Positive Seesaw	57	(12/21)	67	(14/21)	3.05	2.09
Negative Seesaw	37	(7/19)	74	(14/19)	5.00	1.73

*Higher values indicate higher propensity to comment on the petition (Message Processing), sign the petition (Compliance) and to remember information concerning the petition (Information Recall). N, number of participants per condition; M, mean; SD, standard deviation.*

In line with Hypothesis 1, participants in the experimental group (emotional seesaw) tended to be less likely to comment on the petition than participants in the control group (one emotion), χ^2^(1, *n* = 79) = 2.99, *p*_one tailed_ = 0.04, *phi* = -0.22. Participants undergoing a seesaw were also more willing to sign the senseless petition than participants who experienced only one emotion, χ^2^(1, *n* = 79) = 4.59, *p* < 0.05, *phi* = 0.27, confirming Hypothesis 2. Furthermore a univariate ANOVA revealed that as predicted (H3) experiencing an emotional seesaw resulted in impaired information recall, *F*(1,77) = 17.69, *p* < 0.001, $\eta_{p}^{2}$ = 0.19: As seen in **Figure 1B** people in the experimental group (*M* = 3.98; *SD* = 2.14) remembered less information about the petition and the petitionist than participants in the control group (*M* = 5.82; *SD* = 3.98).

To rule out that final emotional state affected results we performed a 2 × 2 between-groups MANOVA (experimental condition: seesaw vs. one emotion; final emotional state: positive vs. negative), using as DVs message processing, compliance, and information recall. As expected, final emotional state had no influence on the dependent variables, Pillai’s Trace = 0.03, *F*(3,73) < 1, *p* = 0.52. Likewise, there was no significant interaction between experimental group and final emotional state, Pillai’s Trace = 0.09, *F*(3,73) = 2.34, *p* = 0.08, which means that neither the direction of the emotional shift in the experimental group, nor the valence of the induced emotion in the control group significantly affected results. As reported above, the influence of experimental condition on the combined dependent variables was significant, Pillai’s Trace = 0.26, *F*(3,73) = 8.73, *p* < 0.001. As the control questions revealed, in hindsight, 85% of participants in the one emotion and 75% in the emotional seesaw groups correctly recalled the purpose of the petition; the difference was not significant, χ^2^(1, *n* = 79) = 1.13, *p* = 0.57, *phi* = 0.12.

### Discussion

Regarding the frequency of compliance with a request, as well as the verbal expression of doubts, the results support earlier findings. Participants displayed impaired message processing, which resulted in higher compliance to sign even a nonsense petition in response to a seesaw manipulation as opposed to the control group were only one emotion was induced (Dolinski and Nawrat, 1998). Additionally, this experiment makes two original contributions: First, it establishes a procedure to investigate the ESP in the laboratory. Second, it is the first to reveal a deteriorating impact of the ESP on information recall.

## Experiment 2

The second experiment was conducted to replicate the findings of Experiment 1. To test additionally whether our manipulation elicits the intended emotions, a manipulation check was included in the experimental script. Therefore a measure of participants’ current emotional state was introduced before and after the manipulation. We expected that the final emotional state in the one-emotion, positive group to be more positive than that in the one-emotion, negative group, with the emotional seesaw groups in between. As in Experiment 1, we further hypothesized that impaired message processing, elevated compliance, and less information recall should be observed in the experimental group (H1-3).

### Participants and Design

A sample of 41 students of a large German university were asked to participate in a common knowledge quiz experiment. One participant from the emotional seesaw group was overlooked by the petitionist and therefore removed from analysis. The one factor (experimental condition: emotional seesaw; *n* = 19 vs. one emotion, *n* = 21) between-subjects design included one additional control factor (final emotional state: positive vs. negative) that should not affect results. There were 19 male and 21 female participants (*N* = 40; *M*_age_ = 22.33, *SD*_age_ = 2.40). Again, the incentive was the chance to win €2.50 and €1 participation endowment.

### Materials and Procedure

Briefly, in ER1, participants’ initial emotional state (T1) was measured on a 5-point Likert-type scale (1 = *very good*; 5 = *very bad*). Subsequently they were randomly assigned to the computer-based condition (answer five questions: easy vs. difficult; expectancy violating vs. expectancy congruent payout scheme) and requested to immediately depart to the second experimental room after answering the quiz. On their way to ER2 they were approached by a male petitionist. In ER2, participants’ current emotional state T2^5^ and informational recall were assessed using questionnaires. Because the male petitionist possessed many distinctive features (e.g., glasses) the *information recall questionnaire* (DV3) was extended from 9 to 14 items. All other details of the procedure were identical to Experiment 1.

### Results

Replicating the results of Experiment 1, fewer participants in the experimental group asked questions concerning the petition than in the control group [31.6% (6 out of 19) vs. 61.9 % (13 out of 21)], χ2(1, *n* = 40) = 2.56, *p*_one tailed_ = 0.05, *phi* = -0.30. Furthermore once again participants who experienced an emotional seesaw more often signed the nonsense petition [52.6% (10 out of 19) vs. 19% (4 out of 21)], χ2(1, *n* = 40) = 3.58, *p*_one tailed_ = 0.03, *phi* = 0.35, and remembered less information about its content and the appearance of the petitionist (*M* = 5.63, *SD* = 2.45 vs. *M* = 8.76, *SD* = 4.78), *F*(1,38) = 6.56, *p* < 0.05, $\eta_{p}^{2}$ = 0.15, than participants of the control group.

To ensure that the manipulation evoked the intended emotions, a repeated-measures ANOVA was conducted to assess the impact of the final emotional state (positive vs. negative) and the experimental group (one emotion vs. emotional seesaw) on participants’ current emotional state prior to (T1) and following the intervention (T2). The manipulation check revealed a significant final emotional state × experimental group × time interaction, *F*(1,33) = 4.80; *p* < 0.05, $\eta_{p}^{2}$ = 0.13. Significant changes in emotional state were reported only in the one-emotion group (positive and negative), interaction: *F*(1,17) = 10.93; *p* < 0.01, $\eta_{p}^{2}$ = 0.39. After answering the easy questions participants’ emotional state improved from *T1* (*M* = 2.09; *SD* = 0.70) to *T2* (*M* = 1.53; *SD* = 0.45), but for participants who were asked to answer difficult questions, the emotional state deteriorated from *T1* (*M* = 2.38, *SD* = 0.52) to *T2* (*M* = 3.08, *SD* = 0.68). As expected no emotional changes from T1 to T2 were found in the seesaw groups, all *Fs* ≤ 1. This means that the manipulation evoked the intended emotions in the control groups, but as intended, in the experimental groups, the final emotional state after the emotional seesaw was similar to that at the beginning of the experiment. The elicited emotions however did not influence results. As in Experiment 1, the multivariate MANOVA showed no statistically significant influence of the induced final emotional state (*F* < 1.57, *p* = 0.22), nor interaction with experimental group on the dependent variables (message processing, compliance, information recall, *F* < 1).

### Discussion

The findings replicate the emotional-seesaw effect demonstrated in Experiment 1. The decrease in message processing and the increase in compliance relative to the control group indicate that a higher inclination toward persuasion that occurred apparently because of shallower information processing. As in Experiment 1, compliance with a nonsense request was the dominant response. Experiment 2 furthermore provided evidence that the procedure elicited the intended emotions. An interesting finding, however, is that the emotions changed in the intended direction in the control group, indicating a successful emotion induction, but remained stable in the ESP groups. This could explain why the direction of the emotional seesaw did not influence findings. Afifi and Metts (1998) claim that an expectancy violation always results in a cognitive arousal and an initiation of a series of interpretations and evaluations that aid an individual in coping with the unexpected outcome. Because the post affective measure was assessed after the walk from one room to the other (not immediately after the manipulation), we believe this to be a sign that during the walk from one room to the other a cognitive appraisal/interpretation took place that restored the affective status quo.

## General Discussion

Contrasting emotions lead to losses in processing efficacy, leaving individuals momentarily vulnerable to social influence attempts (Dolinski and Nawrat, 1998). We were able to confirm these assumptions in a controlled laboratory setting. Individuals subjected to an emotional seesaw did not only display inhibited information processing, as indicated through a lack of verbalizations in response to a senseless request, but also displayed a higher tendency toward its fulfillment. By demonstrating this, the current experiments provide a conceptual replication of Dolinski and Nawrat’s (1998) earlier findings. Our results extend the assumption that contrasting emotions inhibit processing efficiency, showing its detrimental impact on information recall. Finally, we were able to develop and validate an easy to implement and properly counterbalanced procedure to evoke contrasting emotions in a laboratory setting using only a mild form of participant deception (i.e., information omission). Given the clear success of the present replication attempt, it is interesting to consider the possible reason why people yield mindlessly to social influence after a sequence of changed emotions. So far it has been proffered that the ESP evokes mindlessness. This supposition was confirmed in various experiments. First people after an ESP have shown processing deceleration when confronted with perceptual tasks like arithmetical calculations, or emotion detection (Dolinski et al., 2002). Second, Dolinski and Nawrat (1998) showed that after a sequence of changed emotions people tend to accept even dubious reasons when asked for money donation. We were able to confirm this lack of critical cognitive dispute of information when confronting people with a clearly senseless petition. This processing inhibition was visible in the lack of comments and concerns in response to senseless information.

One may argue that overt behavior does not necessarily reflect participants’ current processing mode. However, the debilitated information recall of petition associated information clearly confirms our interpretation. It should be noted that attention was not completely absent, but seemingly disengaged from the ongoing scenario. Later, when asked to recall the wording of the petition, participants were able to do so, but, importantly, many reported to realize its meaning for the first time. Apparently visual (aspects of petitionist) as well as auditory (content of petition) components of the compliance scenario were partially encoded by participants, but not mentally integrated in working memory. This new finding shows that the ESP does not completely impair the appraisal of a situation, only the application of the information in the appraisal.

Dolinski et al. (2002) furthermore managed to show that a mindless state of mind is crucial for compliance to take place. In an experiment they forced emotionally seesawed individuals back to mindfulness which resulted in a decrease of compliance rates to control group levels (Dolinski et al., 2002). Research has shown that people in a mindless processing mode do not invest much thought into deciding how to respond when presented with a request (Langer et al., 1978), rather processing information automatically via mental shortcuts, also called heuristics (Cialdini, 2001). In the present experiments, participants’ tendency to comply with the request could have been based on the heuristic that students typically collect signatures for a good reason. Here, we have to point out that we do not believe that contrasting emotions do intrinsically increase compliance inclination by default, but rather argue that they induce automatic processing, which alters susceptibility to compliance only when compliance promoting heuristic cues are available. Because we created a compliance promoting context in our experiment (i.e., a fellow student collecting signatures for probably a good reason), the extent to which compliant responses were displayed by participants could be elevated as compared to naturalistic settings, where situational cues cannot be controlled to the same extent.

So far, we were able to confirm that contrasting emotions induce mindless processing, which leads to heuristic based compliance. Furthermore these experiments were the first ones to show the ESP’s detrimental impact on information recall. So while there is a solid foundation of research on the ESP and its consequences, the exact mindlessness evoking processes have not been yet elucidated. Because no effects of the direction of the affective shift, in former experiments (Nawrat and Dolinski, 2007) nor ours, on cognitive processing and behavior were found, we argue that increased attention should be paid to other facets of the ESP than the affective dynamic. We hypothesize that the genesis of mindlessness after an ESP does not originate from the affective shift, as so far hypothesized, but can be found in its expectancy violating structure. We assert that each ESP involves a situation that is believed to be true, and this supposition is then abruptly proven inadequate. We propose that the attentional focus on the ESP itself causes a decrease in available processing capacity, which leads to less efficient cognitive processing, congruently inhibiting the onset of mindfulness. Applying this interpretation to our data, one can argue that people exposed to discrepant information elicited by an unexpected payout scheme paid more attention to the inconsistency and hence perceived the subsequent petition scenario less accurately. Future research should test these assumptions.

This experiment has a number of limitations. First, due to the sensitivity of the ESP effect, the manipulation check to corroborate the effectiveness of our emotion induction procedure was included not directly after the manipulation, but after the walk from one room to the other. Based on previous research we know that the inclusion of a measure directly after the ESP would actually undermine the success of the manipulation designed to affect the dependent variables (Dolinski et al., 2002). Second, due to the complexity of the design and its serial approach (25 min per participant) sample size was kept fairly small and the group of participants was quite homogenous (mostly university students), limiting generalizability of findings and impeding insight concerning possible moderating factors such as age or education. Whereas the pattern of findings was replicated across two experiments, a replication with a bigger and more diverse sample is recommended. Nonetheless, the study being conducted in a controlled lab environment also increases its validity.

In closing, our research conceptually replicates findings by Dolinski and Nawrat (1998) and Dolinski et al. (2002), thus confirming that a display of contrasting emotions can impact subsequent cognitive processing and consequently alter susceptibility to persuasive attempts. We were able to not only develop and validate a new ESP induction procedure suitable for a laboratory setting, but also proposed a new possible procedural view underlying the observed consequences. We proffer that delineating the sequential change of emotions may provide critical information on the psychological mechanism by which emotional states affect cognitive processing and executive processes. Within the present context, we hope that the newly developed implementation procedure will guide and inspire future research on contrasting emotions.

## Author Contributions

MK and MS planned and designed the studies. MK conducted the studies, analyzed the data, and wrote the first draft that both authors revised. MK and MS approved the final version of the paper for submission.

## Conflict of Interest Statement

The authors declare that the research was conducted in the absence of any commercial or financial relationships that could be construed as a potential conflict of interest.

## Footnotes

1 For the sake of clarity, throughout the paper we use the terms *emotional seesaw* and *contrasting emotions* interchangeably, referring to experiencing a positive then negative emotional stimulus or vice versa.

2 Participants were only included in analyses if they arrived at at least three (out of five) correct answers in the easy questions condition, or respectively, three wrong answers in the difficult questions condition. All participants reached this threshold.

3 Furthermore, petitionists took notes whether participants stopped when they were approached, whether they listened till the end of the message, and whether they verbally agreed to sign the petition. The first behavioral steps were also treated as possible signs of compliant behavior and analyzed if no significant difference in the number of signatures on the petition was found.

4 No participant correctly identified the research question or experimental hypothesis. Only 22% of participants reported to suspect *ex post* that the petition was part of the experiment. Reported suspicion was not related to any of the dependent measures.

5 Participants’ current emotional state (T2) could not be assessed immediately after the ESP induction because asking cognitively demanding questions after the ESP prompts a shift back to mindfulness, mitigating effects on behavior (Dolinski et al., 2002).
